# Quantifying Personalized Shift-Work Molecular Portraits Underlying Alzheimer’s Disease through Computational Biology

**DOI:** 10.14283/jpad.2024.161

**Published:** 2024-09-03

**Authors:** Y. Xu, G. Zhang, L. Yang, H. Qin, Z. Zhou, Q. Li, H. Liu, R. Wang, Z. Cai, L. Jing, Y. Li, Y. Yao, Z. Gong, P. Yuan, T. Fu, X. Zhao, Tao Peng, Yanjie Jia

**Affiliations:** 1 Department of Neurology, The First Affiliated Hospital of Zhengzhou University, Zhengzhou, Henan, 450052 China; 2 Department of Cardiology, The First Affiliated Hospital of Zhengzhou University, Zhengzhou, Henan, 450052 China; 3 Department of Urology Surgery, The First Affiliated Hospital of Zhengzhou University, Zhengzhou, Henan, 450052 China; 4 Department of Clinical Medicine, Zhengzhou University, Zhengzhou, Henan, 450052 China; 5 Department of Vascular Surgery, The First Affiliated Hospital of Zhengzhou University, Zhengzhou, Henan, 450052 China

**Keywords:** Circadian rhythm, Alzheimer’s disease, shift work, genome-wide association study, genetics

## Abstract

**Background:**

Shift work, the proven circadian rhythm-disrupting behavior, has been linked to the increased risk of Alzheimer’s disease (AD). However, the putative causal effect and potential mechanisms of shift work for AD were still unclear.

**Methods:**

Mendelian randomization (MR) analysis was performed to discover the putative causal effect of shift work for AD. Expression quantitative trait loci (eQTLs) and transcriptome data were integrated to identify genes causally associated with AD from circadian-related genes. An in vitro experiment was also conducted to validate the expression of target genes. Based on the identified genes, a novel integrative program and 4,077 samples from 16 microarray datasets were leveraged to assess the extent of circadian rhythm disruption (CRD), defined as the clock deviation level (CDL).

**Findings/Results:**

Shift work causally increased the risk of AD [odds ratio (OR) = 2.49, 95% CI = 1.79 - 3.19, p = 0.01]. Seven circadian-related genes were causally associated with AD, including CCS, CDS2, MYRIP, NRP1, PLEKHA5, POLR1D, and PPP4C. These genes were significantly correlated with the circadian rhythm pathway. CDL was higher in CRD mice group, shift work group, sleep restriction group, and AD patients compared to control mice group (p = 0.043), non-shift group (p = 0.004), sleep extension group (p = 0.025), and health controls (multiple cohorts, p < 0.05). Additionally, CDL was also significantly correlated with AD’s clinical biomarkers.

**Interpretations/Conclusion:**

By combining GWAS and transcriptome data, this study demonstrated the causal role of CRD behavior in AD, identified the potential target genes in shift work-induced AD, and generated CDL to characterize CRD status, which provided evidence and prospects for disease prevention and future therapeutic interventions.

**Electronic Supplementary Material:**

Supplementary material is available in the online version of this article at 10.14283/jpad.2024.161.

## Introduction

**A**lzheimer’s disease (AD) is a chronic neurodegenerative condition characterized by progressive cognitive decline and neuropsychiatric symptoms (1). The etiology of AD remains obscure with advanced age posing the greatest risk factor for its development (1). Therefore, identifying modifiable risk elements is a critical focus in alleviating the effects of AD on society and medical systems. Shift work refers to a work schedule where employees work outside the conventional 9 a.m. to 5 p.m. timeframe, which could lead to a series of health problems, encompassing psychiatric and metabolic disorders, as well as cognitive impairment (2, 3). Previous works have revealed the detrimental effect of shift work on the occurrence and progression of AD. Findings from UK Biobank indicated that shift work was associated with increased AD incidence compared to non-shift jobs, confirmed in the study of two population-based cohorts encompassing more than 54,000 participants altogether (4, 5). However, shift work was often accompanied by multiple socioeconomic factors, including poor working conditions, long working hours, low income, increased subjective strains and so on (5). Conventional observation studies could not eliminate residual confounding factors and reverse causality completely, which will introduce bias to the result.

Mendelian randomization (MR) analysis was an approach utilizing genetic variants as instrumental variables (IVs) to infer the association between exposure and outcome (6). The inherent properties of genetic variations allocation in MR make it less susceptible to confounding factors, enabling results akin to randomized control trials (RCTs) (7). Thus, MR has emerged as the predominant method to draw causal inferences in the realms of epidemiology and biomedical sciences, which served as the primary approach in our research. Although MR analysis could explore the causal relationship between shift work and AD from a phenotypic level, the mechanism by which shift work caused AD remained obscure. The integration of summary data from genome-wide association study (GWAS) with gene expression data allowed for the identification of expression quantitative trait loci (eQTL). By applying two-sample MR between eQTL and clinical outcome, the potential causal genes could be detected.

As the proven circadian rhythm-disrupting behavior, shift work disrupted the typical patterns of sleep-wake, eating-fasting, and light-dark exposure, all of which served as the most established input signals for the circadian rhythm system (8). Furthermore, circadian rhythm disruption (CRD) has been recognized as a potential mechanism for the occurrence of chronic diseases among shift workers (8). As an internal time-keeping system synchronized with the day-night cycle caused by the Earth’s rotation, circadian rhythm governed various physiological and behavioral processes in organisms (9). The extent of disruption in circadian rhythm could reflect the status of an individual’s health imbalance (10). Despite significant advancements in circadian rhythm research through animal experiments, there was no established indicator for evaluating CRD in the human body. With the progress of genomics and computational biology, defining the status of circadian rhythm could be realized based on the integrative program.

In the current research, we first identified the causal relationship between shift work and AD through MR analysis. Then, by combining differential gene expression analysis and MR analysis at RNA-seq and GWAS levels, we identified target genes both associated with circadian rhythm and causally linked to AD. The target genes were validated via in vitro experiments. Subsequently, based on a novel integrative program, clock deviation level (CDL) was generated to evaluate the degree of CRD at the RNA-seq level. Finally, the robustness and generalization capacity of CDL was tested in multiple GEO cohorts. Our study unveiled the causal relationship between shift work and AD and devised CDL to quantitatively assess the extent of CRD, which identified modifiable risk for AD and provided prospects for AD prevention and potential therapeutic interventions.

## Materials and Methods

**Figure 1:**
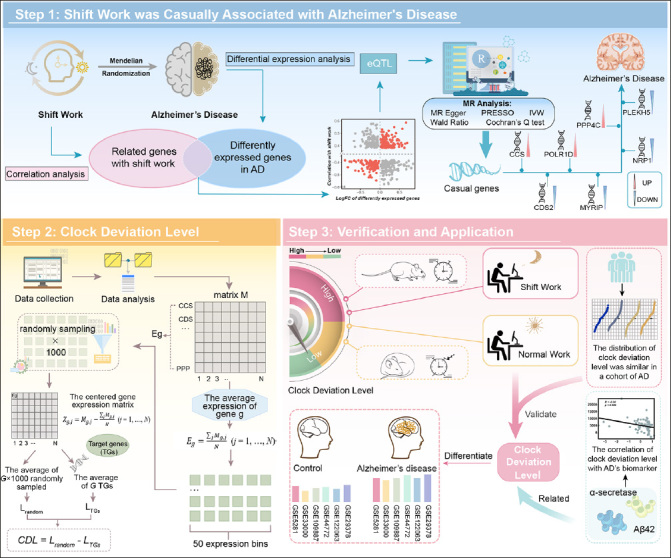
The flowchart of the research progress Step 1 demonstrated the MR analysis between shift work and AD. Step 2 demonstrated that we conducted the CDL based on identified genes. Step 3 demonstrated that we verified the robustness of CDL and explored its clinical application. AD: Alzheimer’s disease; MR: mendelian randomization; IVW: inverse variance weighted; CDL: clock deviation level; eQTL: expression quantitative trait loci.

The general process of this study was exhibited in Figure 1. Details of data and methods utilized were listed as follows.

### Data source and processing

#### GWAS data for shift work

GWAS summary statistics for shift work were available from the UK Biobank involving 263,315 individuals of European ancestry. Shift work was a behavior that could cause CRD, and was defined as “work schedule that falls outside of the normal daytime working hours of 9 AM–5 PM” in UK Biobank. In the original GWAS data of shift work, the frequency was categorized into “Never/Rarely”, “Sometimes”, “Usually” and “Always”, in which “Never/Rarely” was taken as the reference.

#### GWAS data for Alzheimer’s disease

The summary-level data for clinically diagnosed AD was derived from a large GWAS meta-analysis (GWAS ID: ieu-b-2) with 10,528,610 single nucleotide polymorphisms (SNPs). The 63,926 individuals (Ncase = 21,982, Ncontrol = 41,944) in the dataset were also of European ancestry.

#### eQTL dataset

Cis-eQTLs of genes were retrieved from eQTLGen (https://eqtlgen.org/), a gene expression-based meta-analysis GWAS data obtained from 31,684 healthy Europeans.

#### Transcriptional data

The gene expression data were all retrieved from Gene Expression Omnibus (GEO, https://www.ncbi.nlm.nih.gov/geo/). In summary, 4,077 samples encompassing 2,241 AD samples and 1,836 health controls from 16 microarray datasets were utilized in this research, including GSE106241, GSE109887, GSE118553, GSE122063, GSE131617, GSE13214, GSE140829, GSE29378, GSE33000, GSE36980, GSE44772, GSE48350, GSE5281, GSE84422_GPL96, GSE84422_GPL97, and GSE84422_GPL570. The gene expression data of cardiomyocyte-specific Bmal1 knockout (CBK) mice was obtained from GSE43073. The RNA-seq data of shift work mice, including day shift work and night shift work was from GSE124870. The human blood transcriptome following sleep extension and sleep restriction was from GSE82114. Detailed information of GEO datasets was described in Supplementary Table 1.

#### Clinical data collection of the inhouse cohort

This retrospective cohort study was approved by the Ethics Committee of Zhengzhou University (Approval No. 2023-KY-0741-003). Written informed consent was obtained from all participants. We included 45 patients diagnosed with AD according to international diagnostic criteria and 12 healthy participants without cognition impairment. These patients were recruited from the Department of Neurology at the First Affiliated Hospital of Zhengzhou University between January 2023 and January 2024. Clinical data were collected for each patient, including demographic information, disease duration, and clinical presentation. All AD patients underwent magnetic resonance imaging (MRI) to assess hippocampal volume and completed the Pittsburgh Sleep Quality Index (PSQI) and Morningness Eveningness Questionnaire - Self Assessment version (MEQ-SA) to reflect their circadian rhythm performance and preferences of participants.

### Mendelian randomization analysis

As we know, conventional observation studies could not eliminate residual confounding factors and reverse causality completely, which could introduce bias to the result. However, MR analysis is an approach that assesses the causal relationship between exposure factors and outcomes utilizing SNPs that strongly correlate with exposure factors as IVs, drawing causal inferences in the realms of epidemiology and biomedical sciences. Thus, MR analysis was conducted as the main approach to assess the relationships in the current study. In the first step of MR analysis, the GWAS data of shift work was represented as exposure and GWAS data of AD was represented as outcome. In the second step of MR analysis, the cis-eQTLs of candidate genes were taken as exposure factors and GWAS data of AD was taken as outcome.

To verify the stability of the results, sensitivity analyses were conducted. Firstly, MR Egger, weighted median, simple mode, and weighted mode were performed if available. Secondly, leave-one-out was utilized to identify the effect on the outcome driven by a single genetic variant. Furthermore, Cochran’s Q statistic was calculated to check heterogeneity between instrumental variables. Egger regression was performed to check directional pleiotropy on outcome and Mendelian Randomization Pleiotropy RESidual Sum and Outlier (MR-PRESSO) test was performed to check overall horizontal pleiotropy among all SNPs. Finally, reverse MR analysis was further performed to avoid the causality effect of outcome on exposure.

### Identification of circadian-related genes causally associated with AD

To unveil genes potentially act in shift work-induced AD, which is both associated with circadian rhythm and also contributed causal roles in the development of AD, we integrated GWAS and transcriptome data and took three steps as follows:

1. Taking the intersection of genes associated with shift work and those differentially expressed in AD, only genes with a consistent trend of upregulation or downregulation in both conditions were included as candidate genes.
2. MR analysis between cis-eQTLs and AD was conducted to evaluate the causal effects of candidate genes on AD. Only genes with FDR < 0.1 were selected.
3. Gene set variation analysis (GSVA) was performed to obtain the enrichment score of the “GOBP_CIRCADIAN_RHYTHM” pathway. Then, we calculated the correlation between the expression of genes obtained from the second step and the enrichment score. Only genes significantly correlated with the circadian rhythm pathway were retained as target genes (TGs).

### Validation of the targeted genes in inhouse cohort

The expression levels of target genes were validated through in vitro experiments. Initially, we compared the relative mRNA levels of these genes using quantitative real-time polymerase chain reaction (qPCR). The primer sequences of target genes were listed in Supplementary Table 2. Additionally, to detect and quantify the levels of the corresponding proteins, phosphorylated tau 181 (p-tau181), and amyloid beta 1–42 (Aβ1-42), enzyme-linked immunosorbent assays (ELISA) were conducted. Detailed descriptions of these methods can be found in the Supplementary Methods.

### Clock deviation level generated from the novel integrative program

A novel algorithm that deciphered the variation in signal-to-noise ratio among target genes (TGs) based on transcriptional data was utilized to assess the degree of circadian rhythm disruption in each sample. This metric, termed clock deviation level (CDL), was calculated through three steps (10–12):

1. Bulk RNA-seq and microarray data were normalized as expression matrix M. In M, the average expression (E_g_) of gene g across N samples was defined as:
  $$E_{g}={\Sigma_{j}M_{g,j}\over {N}}\ (j=1,\ldots, N)$$
  According to E_g_, all genes were categorized into 50 expression bins. And times of appearance of TGs (their frequency within each bin) were designated as B_TGs_. Then, a random sampling strategy underwent repetition 1000 times was employed. Random signature genes were then selected from each bin based on the random sampling B_TGs_, which ensured random signature genes (G) and TGs consistent in number.
2. The centered gene expression matrix (Z_g,j_, central expression of gene g in sample j), which represented data without excessive migration signal, was calculated as follows:
  $$Z_{g,j}=M_{g,j}-{\Sigma_{j}M_{g,j}\over {N}}\ (j=1,\ldots , N)$$
3. The clock deviation level (CDL), which conformed to a mixture of normal distribution and deciphered the abundance of TGs, was calculated as follows:
  $$CDL=L_{random}-L_{TGs}$$

L_random_ was a random score equaled to the average of G × 1000 randomly sampled features above and L_TGs_ was the average of G TGs via the centered expression data for each sample.

### Validation of clock deviation level

The CDL was compared between the normal and CRD groups to validate its ability to reflect circadian rhythm disruption. Furthermore, CDL comparisons were conducted among the normal, day shift work, and night shift work groups, along with the sleep extension and sleep restriction groups, providing a comprehensive analysis of CDL across diverse circadian patterns. Finally, we explored the CDL in AD and normal groups, shedding light on correlations between CRD and AD.

### The robustness and stability of the clock deviation level

To test the stability of clock deviation level across various datasets, individual and average CDL within 16 GEO cohorts were calculated. The overall mean CDL for all 16 cohorts was also identified. Subsequently, we compared the collective mean of 16 cohorts with the mean of the individual cohorts. Furthermore, the distribution of CDL by the cutoff of quartiles and means in 16 cohorts was exhibited.

### Association between clock deviation level and biomarkers of AD

To explore the impact of CRD on molecular processes associated with AD progression, we delved into the relationship between CDL and key indicators of AD, including α-secretase activity and Aβ-42 level, both of which exhibited a decrease in AD patients.

### Statistical analysis

Detailed methods were described in Supplementary Methods. All data processing and analysis were performed in R 4.3.1 software. The mendelian randomization was conducted via the TwoSampleMR package. For comparisons of continuous variables, the Wilcoxon test was used for two groups, and the Kruskal-Wallis test was used for three or more groups. Pearson and Spearman correlation analyses were performed to determine correlations between variants. False discovery rate (FDR) adjustment was utilized for multiple comparison corrections. All statistical P values were two-sided. P < 0.05 was considered as statistically significant.

## Results

### Causal effects of shift work on Alzheimer’s disease

**Figure 2:**
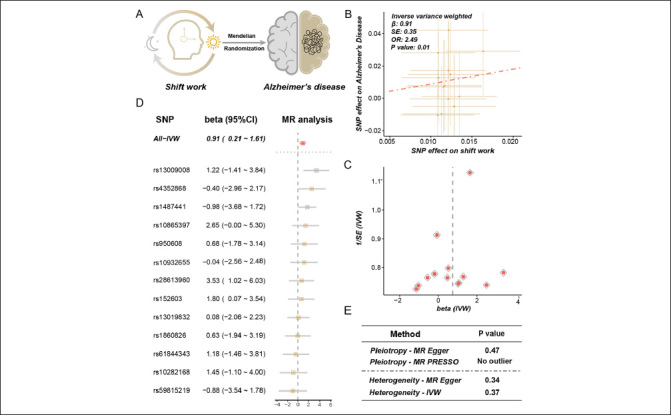
Results of mendelian randomization between shift work and Alzheimer’s disease A. Mendelian randomization (MR) analysis was conducted between shift work (exposure) and Alzheimer’s disease (AD) (outcome). B. The effects of the causal association between shift work and AD. The points represented SNPs and the slope of the line represented the estimated MR effect. C. The funnel plot of the MR analysis result. D. The detailed forest plot of sensitivity tests by leave-one-out analysis. E. The result of pleiotropy and heterogeneity for MR analysis

We conducted MR analysis to identify the causal effects of shift work on AD (Figure 2A). As described in methods, it was observed that 36 SNPs were significantly and independently associated with shift work. To avoid potential weak instrument bias, we calculated the F-statistic for IVs, and only 15 IVs with F-statistic exceeding 10 were included in the final analyses. The F-statistics of these IVs were illustrated in Supplementary Table 3.

Results of MR analysis showed that shift work was causally associated with AD [odds ratio (OR) = 2.49, 95% CI = 1.79 – 3.19, p = 0.01, method = IVW] (Figure 2B, C). The effect of each single SNP was demonstrated in Figure 2D. The sensitive analysis consistently indicated a suggestive and positive causal association with AD, with detailed information provided in Supplementary Table 4. To investigate and correct pleiotropy for the outcome, we conducted Egger regression and MR-PRESSO. As demonstrated in Figure 2E, no directional and horizontal pleiotropy was found (p = 0.47, method = MR Egger; no outlier was found, method = weighted median). The heterogeneity was tested via Cochran’s Q statistic and no heterogeneity was observed (p = 0.34, method = MR Egger; p = 0.37, method = IVW) (Figure 2E). Finally, the MR analysis of AD on shift work was performed to avoid a reverse causal effect, and no evidence was identified for the causal effect of AD on shift work (OR = 0.997, 95% CI = 0.991 – 1.003, p = 0.34, method = IVW) (Supplementary Table 5).

### Identification of circadian-related genes causally associated with AD

**Figure 3:**
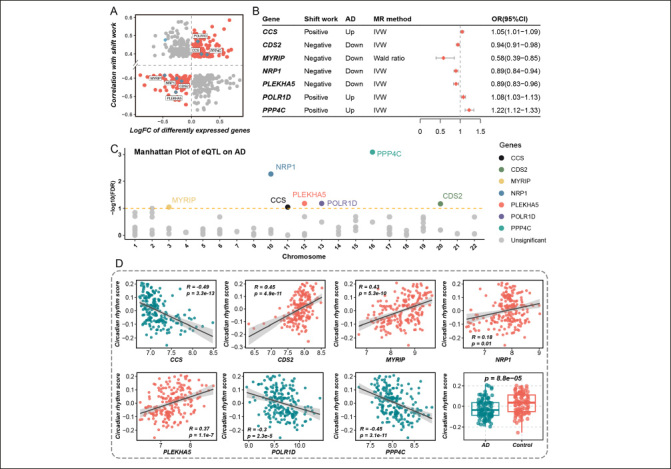
The identification of target genes both correlated with circadian rhythm and causally associated with Alzheimer’s disease A. The intersection of genes is both associated with shift work and differently expressed in Alzheimer’s disease (AD). The X-axis represented the logFC of differently expressed genes between AD and health control. The Y-axis represented the correlation coefficient between gene expression and shift work. B. The result of mendelian randomization (MR) between expression quantitative trait loci (eQTL) of genes and AD. Positive and negative represented genes positively and negatively correlated with shift work respectively. Up and down represented genes upregulated and downregulated in AD compared to health control. IVW represented the inverse variance weighted method. The OR (95%CI) represented the MR result between eQTL of genes and AD. C. The Manhattan plot of eQTL effect of genes on AD. The X-axis represented the chromosome position of genes. The Y-axis represented the -log10(FDR) of the MR result. D. The correlation between the expression of target genes and enrichment score of the circadian rhythm pathway. The last box plot represented the comparison of the enrichment score of the circadian rhythm pathway in AD group and health control group

Shift work was known as the circadian rhythm-disrupting behavior. It has been proven to disturb our internal circadian rhythm and synchronization with light-dark cycles, leading to a variety of disorders within the physical environment. Preceding cohort studies have revealed the link between shift work and dementia (4, 5, 13), and their causal relationship has been substantiated through the MR analysis above. To uncover dysregulated genes in circadian rhythm-disrupting behavior which also contributed to the occurrence of AD, we conducted a comprehensive analysis through three steps. Firstly, we identified the differently expressed genes between AD and health control, in which 464 genes overlapped with dysregulated genes linked to shift work (Figure 3A). Only 220 genes showed a consistent trend of upregulation or downregulation in both conditions and were listed as candidate genes (Figure 3A). Then, MR analysis between the expression of candidate genes (cis-eQTLs) and AD was performed. As displayed in Figure 3B, seven genes were identified causally associated with AD, including CCS (OR = 1.05, 95% CI = 1.01 – 1.09, method = IVW), CDS2 (OR = 0.94, 95% CI = 0.91 – 0.98, method = IVW), MYRIP (OR = 0.58, 95% CI = 0.39 – 0.85, method = Wald ratio), NRP1 (OR = 0.89, 95% CI = 0.84 – 0.94, method = IVW), PLEKHA5 (OR = 0.89, 95% CI = 0.83 – 0.96, method = IVW), POLR1D (OR = 1.08, 95% CI = 1.03 – 1.13, method = IVW), PPP4C (OR = 1.22, 95% CI = 1.12 – 1.33, method = IVW) (Supplementary Figure 1). The β value of MR analysis was additionally noted in Supplementary Table 6. Furthermore, the positive and negative effects of genes on AD via MR were consistent with their effects as observed in the differential expression analysis (Figure 3B). We also illustrated the MR results of all 220 candidate genes in Manhattan plot and the seven genes that met the selection criteria were highlighted (Figure 3C). Finally, we calculated the correlation between the expression of these seven genes and the enrichment score of the circadian rhythm pathway. All seven genes significantly correlated with the circadian rhythm pathway and were defined as target genes (TGs) (Figure 3D).

### Validation of targeted genes in inhouse cohort

**Figure 4:**
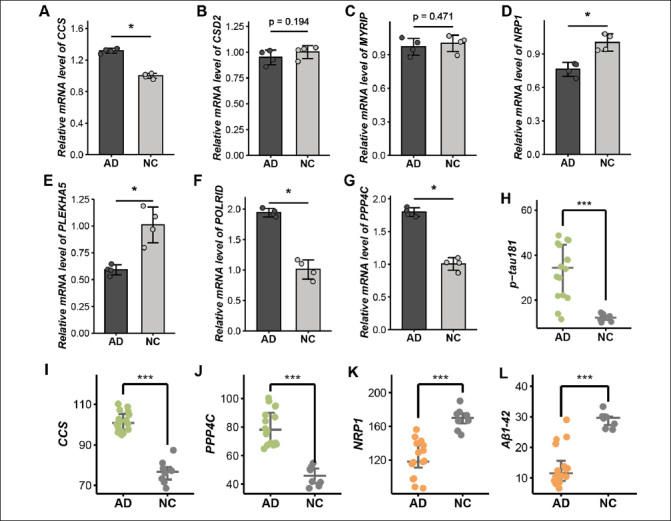
Validation of target genes in vitro experiments. A-G. The relative mRNA level of seven target genes including CCS (A), CDS2 (B), MYRIP (C), NRP1 (D), PLEKHA5 (E), POLR1D (F), and PPP4C (G) via qPCR. H-L. The expression level of p-tau181 (H), CCS (I), PPP4C (J), NRP1 (K), and Aβ1-42 (L) by ELISA

We compared the expression level of seven target genes between AD and HC via qPCR. Consistent with our findings from computational biology analysis, CCS, POLRID, and PPP4C exhibited elevated levels in AD. Conversely, NRP1 and PLEKHA5 were significantly lower in AD. Although CSD2 and MYRIP did not reach statistical significance, they exhibited a trend toward reduced expression (Figure 4A-G; Supplementary Table 7).

Furthermore, we conducted ELISA to assess the protein levels corresponding to the target genes. The result illustrated a marked elevation in the level of p-tau181 and expression of CCS and PPP4C in the AD group compared to HC (Figure 4H-J). While the levels of Aβ1-42 and NRP1 were significantly lower in AD, confirming our previous findings (Figure 4K, L; Supplementary Table 8). These results further substantiate the consistency of molecular alterations at both the gene and protein levels.

### Insight into the association between circadian rhythm performance with AD biomarkers from inhouse cohort

To further explore the association between circadian rhythm and AD, we leveraged the MEQ score to reflect the diurnal preferences and sleep-wake patterns. The baseline of characteristics was demonstrated in Supplementary Table 9. We found that people with evening type demonstrated smaller hippocampus size (Supplementary Table 10). The PSQI score reflected the sleep quality of participants, with a higher score representing worse sleep quality. The correlation analysis found that a higher PSQI score was associated with smaller hippocampus size, higher p-tau181 level, and lower Aβ1-42 level (Supplementary Table 10). Taken together, the results implied a potential correlation between CRD extent and the severity of AD. However, the correlation between the questionnaire score and the degree of CRD has not been confirmed, and there was currently no method that could exactly reflect the degree of CRD. To establish an indicator for evaluating CRD in the human body, we utilized a novel integrative program that constructed scores representing the level of clock deviation.

### The construction and robust performance of clock deviation level generated from a novel integrative program

**Figure 5:**
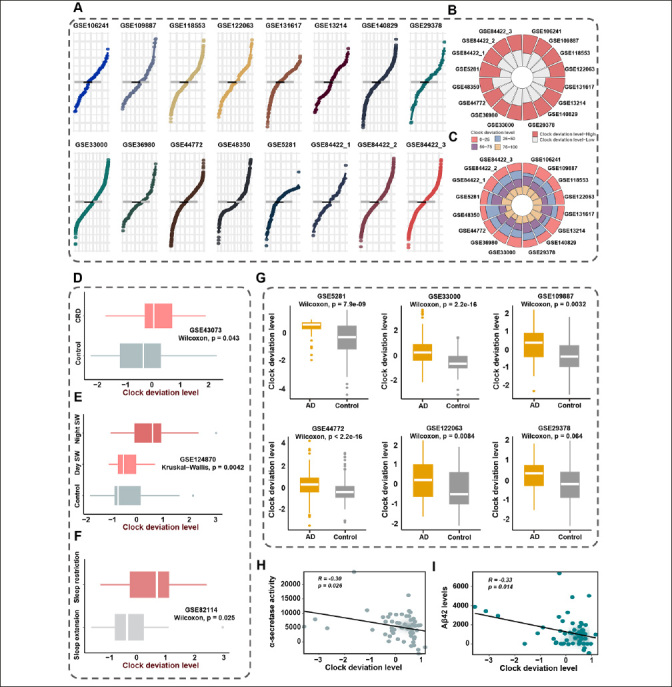
Clock deviation level could reflect the extent of circadian disruption and demonstrated robustness and stability in multiple cohorts A. The distribution of clock deviation level (CDL) exhibited similarity across 16 cohorts. The grey line represented the mean CDL of 16 cohorts. The black line represented the mean CDL of each cohort. B. The circle diagram demonstrated the proportions of samples stratified according to median CDL. Samples were divided into CDL-high and CDL-low groups based on the median score. C. The circle diagram demonstrated the proportions of samples using quartiles as cutoffs. Samples were divided into four groups based on the quartiles of CDL. D. The comparison of CDL between CRD mice group and health control group in GSE43073. E. The comparison of CDL among night shift work group, day shift group, and non-shift work group in GSE124870. Night SW represented night shift work group. Day SW represented day shift work group. Control represented non-shift work group F. The comparison of CDL between sleep restriction group and sleep extension group in GSE82114. G. The comparison of CDL between AD group and health control in multiple cohorts. H. The correlation between CDL and α-secretase activity. I. The correlation between CDL and soluble Aβ42 level

Seven target genes were included in the integrative program to generate clock deviation level (CDL), which demonstrated generalization capacity across multiple cohorts and high discrimination power for AD and health controls. As depicted in Figure 5A, the distribution of CDL exhibited similarity across 16 cohorts comprising over 4000 samples. Additionally, the samples were stratified into CDL-high and CDL-low groups based on the median score. The consistency of group proportions was observed across multiple cohorts, a pattern similarly noted when using quartiles as cutoffs (Figure 5B, C). In conclusion, the generalization capacity of CDL was robust.

To evaluate the reliability of CDL in capturing CRD, we conducted validation in three cohorts, GSE43073, GSE124870, and GSE82114. In the result from GSE43073, CDL was notably higher in CRD mice compared to controls, highlighting the efficacy of CDL in reflecting CRD (Figure 5D). Additionally, the analysis of GSE124870 revealed a progressively increasing trend in CDL across the control group, day shift work group, and night shift work group, consistent with the degree of circadian clock disruption (Figure 5E). We further compared CDL in the sleep extension group and sleep restriction group to assess whether CDL could detect CRD induced by insufficient sleep duration. As anticipated, the sleep restriction group exhibited significantly higher CDL than the sleep extension group (Figure 5F).

Given the evident association between CRD and AD, we conducted the comparison of CDL between individuals with AD and healthy controls. As illustrated in Figure 5G, CDL consistently distinguished between healthy controls and AD, with AD individuals exhibiting higher CDL. In summary, CDL functioned as a metric that not only reliably assessed the extent of CRD but also exhibited differences between AD and normal conditions.

### The biological significance of target genes and CDL

To explore the biological significance of target genes and CDL, we further performed functional analysis. Firstly, we performed the protein-to-protein interaction analysis of identified genes and found that these genes acted in clusters to produce reciprocal effects (Supplementary Figure 2A). Then we conducted an over-representation analysis (ORA) of these genes. Notably, the result demonstrated a strong correlation with several key processes, such as positive regulation of establishment of protein localization to telomere (Supplementary Figure 2B). Furthermore, to elucidate the characteristics of CDL, we explored its underlying mechanisms in depth (Supplementary Figure 3A-D). The CDL score demonstrated strong correlations with several pathways, including immune receptor activity, complementary activation, inflammatory response and so on, which indicated that CDL may affect the immune response and inflammatory pathways to affect the incidence of AD.

### Association between CDL and clinical markers

To assess the clinical interpretability of CDL, we performed the correlation between CDL and clinical markers of AD. The results, illustrated in Figure 5H, revealed a noteworthy negative association between CDL and α-secretase activity (r = −0.30; p = 0.026), a secreted enzyme that prevents the production of amyloid-beta (Aβ) (14). Furthermore, our investigation uncovered an additional negative association between CDL and soluble Aβ42 level (r = −0.33; p = 0.014), which was decreased due to its aggregation to form poorly soluble oligomers in AD (Figure 5I). These findings collectively underscored the significant link between CDL and the clinical application of AD prevention.

## Discussion

By applying MR analysis, we found a causal, detrimental effect of shift work on AD. We also identified seven putative causal circadian-related genes for AD, shedding light on the underlying mechanism connecting gene expression influence by shift work to the development of AD. Employing a novel integrative program, we developed clock deviation level (CDL) as the metric to represent the status of circadian rhythm disruption (CRD), which was significantly higher in CRD groups compared to control groups. Furthermore, CDL also demonstrated a higher level in AD patients than health control in multiple cohorts. This study bridges the gap in our understanding of the relationship between circadian rhythm-disrupting behavior and AD, providing valuable insights into the genetic aspects that contribute to the prevention and potential intervention of AD.

Previous research has illuminated the correlation between shift work, particularly night shifts, and an increased risk of dementia (4, 5, 13). A recent meta-analysis integrated findings from five studies in a quantitative synthesis, bolstering the evidence that shift work contributes to a higher likelihood of developing dementia (15). Additionally, a study conducted within the Danish Nurse Cohort shed light on the cumulative impact of prolonged night shift work (16). Individuals engaged in night shifts for more than 6 years demonstrated a notably elevated incidence of dementia compared to those with less than 1 year of night shift experience. This suggested that the duration of engagement in shift work may play a crucial role in the heightened risk of dementia. Shift work was often accompanied by multiple socioeconomic factors, including poor working conditions, long working hours, low income, and increased subjective strains5. Previous research about shift work was focused on the observational study, which may not fully consider these confounding. To refrain from bias due to unobserved confounding, we conducted MR to estimate an exposure-outcome relation between shift work and AD. Our findings strongly supported a causal link between shift work and AD, underscoring the imperative of avoiding shift work to reduce the risk of AD.

Although shift work could contribute to multiple disruptions physically, the primary culprit behind dementia among shift workers was likely to be circadian rhythm disorders (5). Shift workers experienced perturbative light-dark cycle, eating-fasting cycle, and sleep-wake cycle, which were the established input signals for the circadian rhythm system. The common routine among shift workers involved displacing dark exposure, fasting, and sleeping to the daytime while shifting light exposure, eating, and wakefulness to nighttime, which did not readily adapt to the circadian system (17). Furthermore, a less robust circadian rhythm appeared to be the risk factor for dementia (18).

Circadian rhythm was derived from the network of a master oscillator in the suprachiasmatic nucleus (SCN) and peripheral oscillators, which synchronized with environmental changes induced by the Earth’s rotation (19). Located in the anterior hypothalamus, SCN and its afferent and efferent connections consist the circadian system anatomically. In addition to the main clock in SCN, the peripheral oscillators possessed their circadian clocks, which could be regulated not only autonomously but also synchronized by signals from SCN. The SCN could communicate with peripheral oscillators via neurotransmitters and humoral signals, such as γaminobutyric acid (GABA), cardiotrophin-like cytokine factor 1, transforming growth factor-α and prokineticin receptor 2 (20), which ensured synchronized activity in different brain regions and peripheral tissues. The SCN received input signals related to the light-dark cycle from the retina and was also entrained by variations in light and darkness. An altered light-dark cycle under shift work conditions could perturb the coordinated circadian function caused by SCN and lead to profound effects on the brain. Circadian rhythm regulated the hippocampal-dependent learning and misaligning rhythms in mice demonstrated desynchronized oscillation of clock genes between hippocampus and SCN, leading to impairments in memory and learning (21). Ablation of critical neurons in SCN and disrupt the circadian patterns of neuronal activity were observed in AD patients, which may contribute to their impaired behavioral circadian function and cognition (18).

At the molecular level, the circadian rhythm was controlled by the core circadian clock proteins (18). The conserved circadian clock mediated daily oscillations of the genome in a cell-autonomous manner via interlocking transcriptional-translational feedback loop (20). Under pathological conditions including shift work, the DNA binding activity and expression of core circadian clock genes could be altered, which led to impaired brain health. Previous research on BMAL1-deficient mice demonstrated damaged capacity of learning and memory, impaired brain functional connectivity, synaptic degeneration, and altered hippocampal neurogenesis in these mice compared to mice with natural rhythm (18). These findings suggested that disruptions in the circadian rhythm may contribute to the development of dementia. Furthermore, studies have shown that the diurnal rhythmic expression pattern of clock genes, including ARNTL, PER1, and CRY1 was disrupted in individuals with AD, indicating a connection between alterations in circadian rhythm and the pathophysiology of AD (20).

Although numerous studies have explored the correlation between circadian rhythm and AD, the underlying molecular mechanisms remain elusive. To explore the putative genes causing AD, MR analysis between the eQTL of candidate genes and AD was performed. Seven genes were identified associated with both circadian rhythm and AD, including CCS, CDS2, MYRIP, NRP1, PLEKHA5, POLR1D, and PPP4C.

CDP-diacylglycerol synthase 2 (CDS2) was associated with lipid metabolism and was downregulated in shift workers (22). It played a crucial role in the phospholipid synthesis pathway of cell membranes, influencing the composition and structure of these membranes (22). Although there were no studies have explored the relationship between CDS2 and AD, we could propose the protective role of CDS2 in AD. Deficiency in CDS2 has been observed to compromise the lipid composition, morphology, and function of mitochondria, factors closely linked to the development of AD (23). Further research in this area could provide valuable insights into the potential therapeutic implications of CDS2 in mitigating AD-related pathologies. Myosin VIIA and Rab-interacting protein (MYRIP) was a gene involved in the circadian pathway, which was negatively associated with shift work in our current research (24). Previous GWAS research has demonstrated that variants in the MYRIP gene have been implicated in age-related macular degeneration and cognitive function, which was consistent with its protective role in AD (24). Neuropilin-1 (NRP1) was lowly expressed in shift workers and could encode a non-tyrosine kinase surface glycoprotein involved in various critical biological processes, including the regulation of angiogenesis, and the maintenance of mitochondrial and iron homeostasis (25). Impaired mitochondrial function was an important manifestation in AD patients. Focal iron deposition has been identified in amyloid plaques in the brains of AD patients through MRI, and the interaction between iron and the Aβ peptide may serve as a critical regulator in the formation of mature amyloid plaques (26). Furthermore, the administration of iron chelator deferoxamine to NRP1-deficient endosomal cells could restore mitochondrial function and rescue senescent phenotype (27), elucidating the promise of NRP1 as a therapeutic target for AD. Pleckstrin Homology Domain Containing A5 (PLEKHA5) protein was found anchored to postsynaptic density by super-resolution microscopy, indicating it may play an essential role in brain development (28, 29).

Copper chaperone for superoxide dismutase (CCS) was an important part of the redox system, delivering copper to superoxide dismutase 1 (SOD1) and specific cellular destinations (30). Furthermore, CCS also functioned as a potential tumor promoter in a variety of cancers and several protein-misfolding diseases, including AD and amyotrophic lateral sclerosis (30–32), which may play an essential role in AD development. RNA polymerase I subunit D (POLR1D) was involved in the synthesis of ribosomal RNA precursors and small RNAs, and its overexpression could lead to the proliferation of CRC cells (33). Protein phosphatase 4 catalytic subunit (PPP4C) was one of the PPPCs family, which was involved in the development of several tumors (34). We found that POLR1D and PPP4C were upregulated in shift workers and causally associated with AD, which has not been illustrated in previous research and requires further exploration.

These seven genes identified through MR demonstrated significant correlations with circadian rhythm and AD, illustrating their potential role in shift work causing AD. Although previous research has explored the deep mechanism of circadian rhythm via experiments in vivo and in vitro, no research assessed the extent of disruptions in the body’s circadian rhythm and their association with AD. To assess the CRD level of individuals, an algorithm that deciphered the variation in signal-to-noise ratio among genes based on circadian-related genes’ expression profiles was utilized (10) and thus CDL was calculated. CDL demonstrated high discrimination between CRD mice and health control, indicating the robustness of CDL in reflecting CRD level. Furthermore, CDL was higher in night shift workers than in day shift workers, indicating a severe circadian rhythm perturbance in night shift workers. We also found that individuals with sleep restriction demonstrated disrupted circadian rhythm in contrast to those with sleep extension, which may partly explain the elevated CDL in shift workers. Shift workers were a vulnerable group often accompanied by sleep restriction and compromised sleep quality, which were detrimental factors in developing CRD2. Additionally, when we calculated CDL in AD group and health control, AD group exhibited higher CDL than health control. This indicated that circadian rhythm was disrupted in AD group, consistent with their lower enrichment score of the circadian rhythm pathway.

CDL was also found negatively associated with α-secretase activity and soluble Aβ42 levels in AD patients. Alpha-secretase was an enzyme that could induce alpha cleavage of amyloid precursor protein (APP), which in turn produced soluble, neurologically friendly proteins (35). This process helped to prevent the formation of beta-amyloid (Aβ), the deposition of which was an important pathological feature in the development of AD. In patients with AD, decreased α-secretase activity resulted in APP being more likely to undergo β-cleavage, generating toxic Aβ proteins (36). Therefore, investigating how to modulate or enhance a-secretase activity to induce the α-cleavage pathway may be a potential strategy for the treatment of AD. Aβ42 was a protein fragment cleaved from APP, which could aggregate to form oligomers and become poorly soluble (37). The abnormal aggregation and deposition of Aβ42 in the brain could form plaques, which was the major pathological feature of AD (38). Aggregation of Aβ42 often represented a decrease in soluble Aβ42, which was associated with poor cognition and smaller hippocampal volume (38). To sum up, the negative correlations were consistent with elevated CDL in AD patients, which intensified the clinical interpretability of CDL.

The current research was limited by several factors. Firstly, the MR analysis between shift work and AD was confined to Europeans, as the GWAS data for other ethnicities were not well developed. However, the occurrence of AD was influenced, in part, by genetic factors, and these influences may vary to some extent across different ethnic groups. Nevertheless, the impact of shift work on populations appeared to be widespread. Therefore, it was imperative to replicate these analyses in other ethnicities to accurately capture the influence of shift work on the development of AD across diverse ethnic backgrounds. Secondly, the absence of a gold standard for assessing the extent of circadian rhythm disruption meant that our CDL could not be compared with findings from previous studies. In the future, direct analysis of circadian biomarkers such as cortisol or melatonin will be essential to refine the measurement of circadian deviation. Lastly, it was essential to validate our findings through experimentation in both cellular and animal models. Subsequent studies will involve conducting experimental research to gain a more profound insight into the development of AD induced by shift work and the underlying mechanisms of circadian rhythm disruption.

In the present study, we revealed the causal association between shift work and Alzheimer’s disease (AD) through Mendelian randomization (MR) analysis, highlighting that shift work has a detrimental impact on the occurrence of AD. We further identified seven genes related to both circadian rhythm and AD through a comprehensive investigation that integrated transcriptome analysis and MR analysis. Based on these target genes, we constructed the clock deviation level (CDL) to quantify the extent of circadian disruption in clinical settings, which also demonstrated higher levels in AD patients. The current research was the first to combine the transcriptome with Genome-Wide Association Study (GWAS) data to explore circadian rhythm-disrupting behavior and AD, which demonstrated public health significance in the realm of AD prevention and offered a broad biological and clinical perspective for future therapeutic interventions.

## Electronic supplementary material

Supplementary material, approximately 826 KB.

## Data Availability

*Data availability:* Public datasets included in the present work were from GEO, IEU Open GWAS project, UK Biobank, and eQTLGen.

## Notes

*Ethics approval and consent to participate:* This study was approved by the Ethics Committee of Zhengzhou University (Approval No. 2023-KY-0741-003). Written informed consent was obtained from all participants.
