# Supplementary material for: Quantifying Personalized Shift-Work Molecular Portraits Underlying Alzheimer’s Disease through Computational Biology
Source: J Prev Alzheimers Dis. 2024 Sep 3;11(6):1721–33. doi: 10.14283/jpad.2024.161 (PMC11573866; doi:10.14283/jpad.2024.161)
Supplement: Supplementary file 1 — Supplementary material, approximately 826 KB. [file 42414_2024_362_MOESM1_ESM.docx]

**Supplementary Materials**

- **Supplementary Methods**
- **Supplementary Figures**
- **Supplementary Tables**
- **STROBE-ME**

**Supplementary Methods**

**Gene expression analysis**

To obtain shift work-related genes, we conducted correlation analysis between gene expression and shift work status. Genes with *p* value < 0.001 were taken as genes associated with shift work. We performed differential analysis of Alzheimer’s disease (AD) patients and normal controls to obtain differentially expressed genes. The differential analysis was conducted via *limma* package.

**GSVA analysis**

Gene set variation analysis (GSVA), is a non-parametric, unsupervised algorithm that calculates the enrichment score for a specific set of genes in each sample without pre-grouping the samples. We performed GSVA to calculate the enrichment score of the “GOBP_CIRCADIAN_RHYTHM” pathway, which was obtained from Gene Ontology (GO) database.

**Mendelian randomization analysis**

Mendelian randomization (MR) analysis in this research conformed to the STROBE-MR Statement^1^ and was based on three major assumptions: (i) the selected IVs are strongly associated with exposure factors. (ii) the selected IVs are independent of confounders. (iii) the selected IVs can only act on outcomes through exposure factors.

To choose the optimal IVs for shift work, GWAS-significant SNPs (threshold was set to 5×10^−6^) were pruned by clumping under a stringent window (r^2^ < 0.001; clumped distance = 10,000 kb) to avoid the linkage disequilibrium (LD). SNPs with minor allele frequency (MAF) less than 0.01 were removed. Furthermore, *F*-statistic was calculated to assess strength of IVs, and only SNPs with *F*-statistic more than 10 were retained. Then the exposure data and outcome data were harmonized and inverse variance weighted (IVW) was utilized to conduct the MR analysis between shift work and AD. For MR analysis between cis-eQTLs of genes and AD, SNPs were selected within 1000 kb upstream and downstream of each gene’s transcriptional region at genome-wide significance (*p* < 5×10^−6^) and were further clumped at r^2^ < 0.1^2^. MR estimates for single SNP were calculated via Wald ratio and IVW was performed in the case of more than one SNP available.

To verify the stability of the results, sensitivity analyses were conducted. Firstly, MR Egger, weighted median, simple mode, and weighted mode were performed if available. Secondly, leave-one-out was utilized to identify the effect on the outcome driven by a single genetic variant. Furthermore, Cochran’s Q statistic was calculated to check heterogeneity between instrumental variables. Egger regression was performed to check directional pleiotropy on outcome and Mendelian Randomization Pleiotropy RESidual Sum and Outlier (MR-PRESSO) test was performed to check overall horizontal pleiotropy among all SNPs. Finally, reverse MR analysis was further performed to avoid the causality effect of outcome on exposure.

**Sleep Characteristics Assessment Using the MEQ-SA**

To assess individual differences in diurnal preferences and sleep-wake patterns, participants completed the Morningness Eveningness Questionnaire - Self Assessment version (MEQ-SA). The MEQ-SA was a validated tool comprising 19 items, which quantified an individual's morning or evening orientation in daily activities. Each item was scored on a scale that varies depending on the question, contributing to a total possible score ranging from 16 to 86. The MEQ-SA was administered during initial study visits, and all responses were collected and analyzed to classify participants into one of three chronotypes: definite morning type (score above 59), moderate morning type (score between 53 and 59), neutral type (score between 42 and 52), moderate evening type (score between 31 and 41), and definite evening type (score below 31).

**Assessment of Sleep Quality**

Sleep quality was assessed using the Pittsburgh Sleep Quality Index (PSQI), a standardized self-rated questionnaire that evaluates sleep quality and disturbances over a one-month time interval. The PSQI consists of 19 individual items, generating seven "component" scores: subjective sleep quality, sleep latency, sleep duration, habitual sleep efficiency, sleep disturbances, use of sleeping medication, and daytime dysfunction. Each component is scored on a scale from 0 to 3, where 3 indicates the greatest dysfunction. The component scores are then summed to produce a global PSQI score, which ranges from 0 to 21, with higher scores indicating poorer sleep quality. A PSQI score greater than 5 differentiates poor sleepers from good sleepers with a diagnostic sensitivity and specificity of 89.6% and 86.5%, respectively, as validated in previous studies. This tool has been extensively used in both clinical and population-based studies and is considered reliable and valid for assessing sleep quality and disturbances.

**RNA extraction and quantitative real‑time polymerase chain reaction (qPCR)**

Total RNA was extracted from plasm of Alzheimer’s disease (AD) patients and health controls (HC) using Trizol following the manufacturer’s protocol. The quality and concentration of RNA were determined by NanoDrop spectrophotometer (Thermo Fisher Scientific, USA). Total RNA was reverse-transcribed using SweScript All-in-One RT SuperMix for qPCR (G3337, Servicebio, China) for complementary DNA (cDNA) synthesis according to the manufacturer’s instructions. Quantitative PCR was performed on a QuantStudio 5 Real-Time PCR System (Applied Biosystems, USA) using 2×Universal Blue Probe qPCR Master Mix (G3327, Servicebio, China). The specific primers used for the amplification of target genes and the reference gene (GAPDH) were listed in Supplementary table 2. The thermal cycling conditions included an initial denaturation at 95°C for 10 min, followed by 40 cycles of 95°C for 15s and 60°C for 1 min. The relative expression levels of target genes were calculated using the 2^(-ΔΔCT) method, normalizing against GAPDH as the internal control.

**Enzyme-Linked Immunosorbent Assay (ELISA)**

Serum samples were collected from AD patients and HC, and stored at -80°C until analysis. Quantitative determination of PPP4C and CCS levels was performed using the Finetest [Human PPP4C(Serine/threonine-protein phosphatase 4 catalytic subunit) ELISA Kit and Human CCS (Copper chaperone for superoxide dismutase) ELISA Kit] following the manufacturer’s protocol. The ELISA Kit of NRP1 and Aβ1-42 were obtained from Elabscience. Samples and standards were prepared according to the kit's instructions and added to the pre-coated ELISA plates provided by the kit. Each well was loaded with 100 µL of either standard or sample in duplicate. The plates were incubated at room temperature to allow the binding of analytes to the coated antibodies. Detection antibody, conjugated with enzyme-labeled reagent, was then added to each well and the plates were incubated for 1 hour at room temperature. Finally, add 100 µL of substrate solution to each well and perform a 20 min colorimetric treatment at room temperature in the dark. The optical density (OD) of each well was measured at 450 nm using a microplate reader. A standard curve was generated using the OD values of the provided standards (SpectralMax M5e). The concentrations of the analytes in the samples were calculated based on this standard curve.

**Association between circadian rhythm performance and preference with biomarkers of AD in inhouse cohort**

The PSQI score could reflect the stability of circadian rhythms and the MEQ score could reflect the preference of circadian rhythms, which were elaborated in the Supplementary Methods. We examined the correlations between the PSQI and MEQ scores and several AD biomarkers, including hippocampal volume, p-tau181, and Aβ1-42, which aimed to explore the relationships between circadian rhythm performance and preference with biomarkers of AD.

**The novel integrative program generating clock deviation level**

Compared to the expression of a single gene, gene modules had a stronger ability to resist noise interference, providing more coherent signals. To calculate the overall expression (OE) of gene modules or features, we employed a method that filtered technical variations and highlighted biologically meaningful patterns. This approach was based on the following concepts: the measured expression of a specific gene was related to its true expression (signal) but also contains technical (noise) components. The latter may result from various random processes during the capture and amplification of gene transcripts, sample quality, and variations in sequencing depth. Among other factors, the signal-to-noise ratio also depended on the abundance of gene transcripts. We therefore computed the OE of gene signatures in a way that accounted for the variation in the signal-to-noise ratio across genes and samples.

1. Skrivankova VW, Richmond RC, Woolf BAR, et al. Strengthening the reporting of observational studies in epidemiology using mendelian randomisation (STROBE-MR): explanation and elaboration. *BMJ (Clinical research ed)* 2021; **375**: n2233.

2. Zhang J, Chen Z, Pärna K, van Zon SKR, Snieder H, Thio CHL. Mediators of the association between educational attainment and type 2 diabetes mellitus: a two-step multivariable Mendelian randomisation study. *Diabetologia* 2022; **65**(8): 1364-74.

**Supplementary Figure**

- Supplementary Figure 1
- Supplementary Figure 2
- Supplementary Figure 3


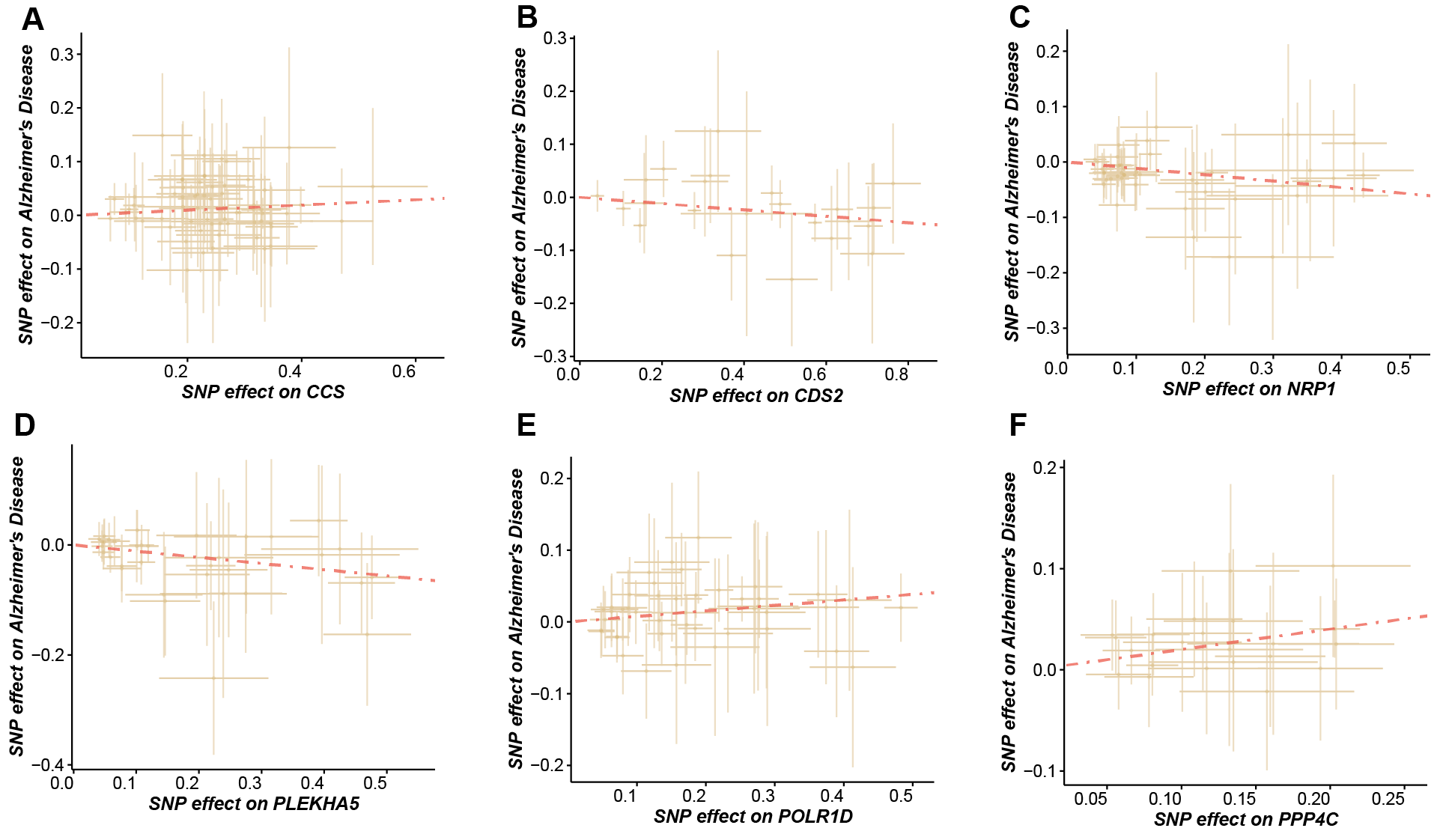


**Supplementary Figure 1: The effects of causal association between genes and AD.** The points represented SNPs and the slope of line represented the estimated MR effect.


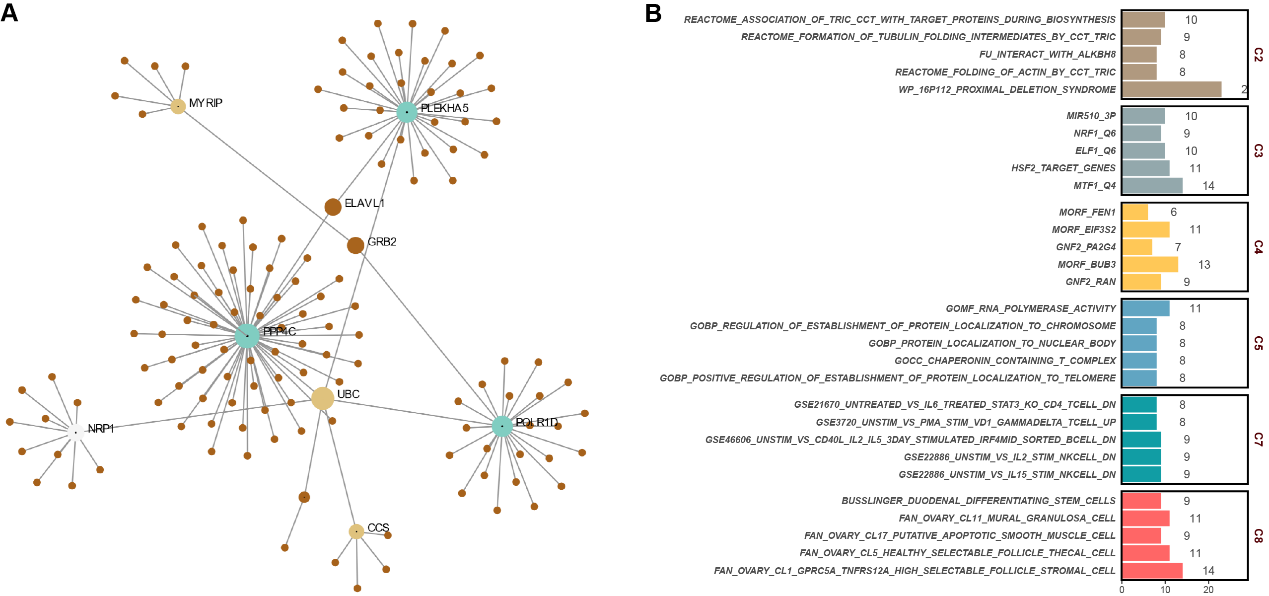


**Supplementary Figure 2: The biological significance of target genes. A.** The protein-to-protein interaction analysis of target genes. **B.** The over-representation analysis of target genes.


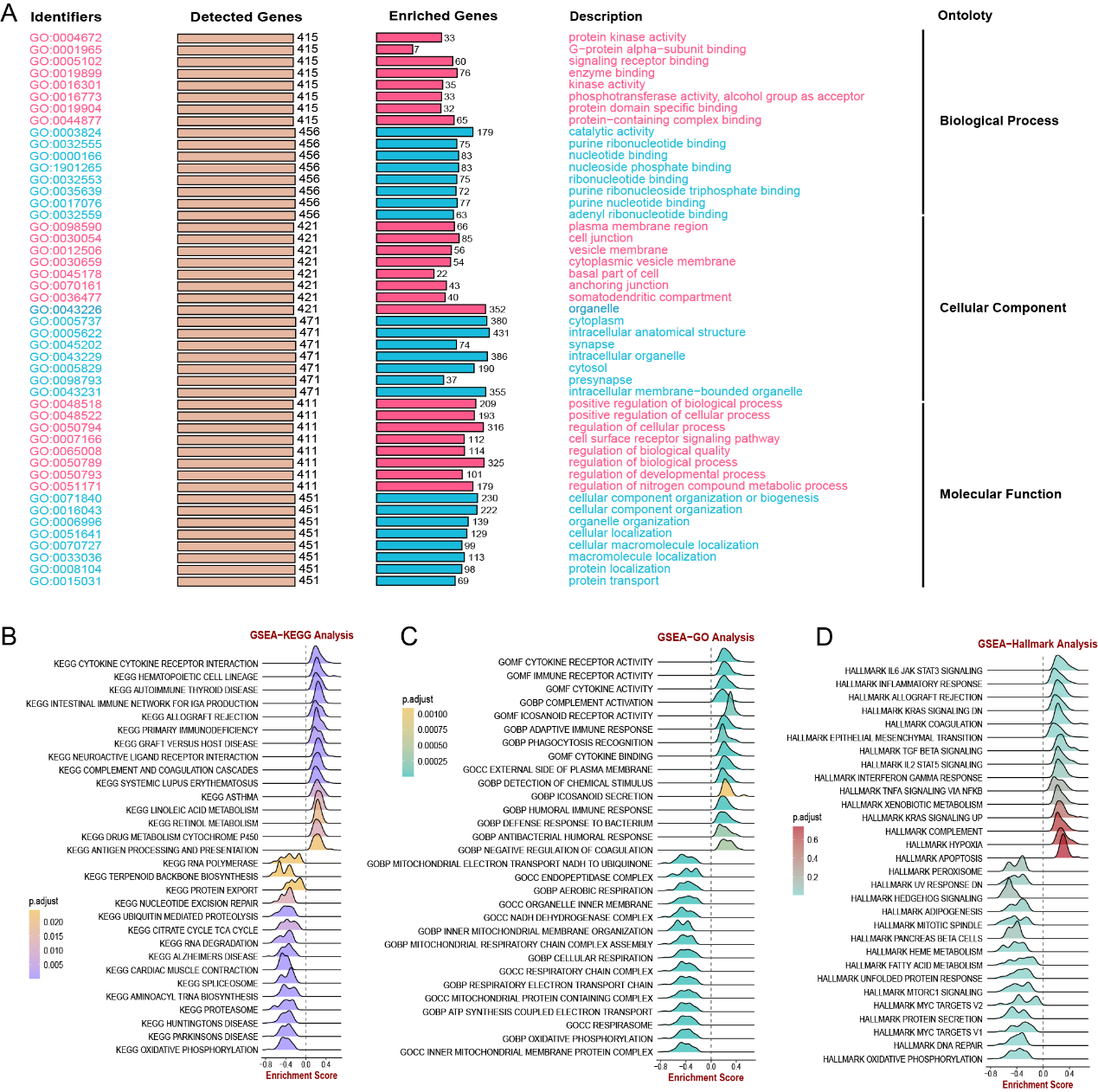


**Supplementary Figure 3: The biological significance of CDL. A.** The GO result of CDL-related genes. **B-D.** The GSEA result of different pathways of CDL-related genes.

**Supplementary Table**

- **Supplementary Table 1**
- **Supplementary Table 2**
- **Supplementary Table 3**
- **Supplementary Table 4**
- **Supplementary Table 5**
- **Supplementary Table 6**
- **Supplementary Table 7**
- **Supplementary Table 8**
- **Supplementary Table 9**
- **Supplementary Table 10**

**Supplementary Table 1: Detailed information of GEO cohorts utilized in analysis.**

| GSE | Sample | AD | CTL |
| --- | --- | --- | --- |
| GSE140829 | 453 | 204 | 249 |
| GSE118553 | 267 | 167 | 100 |
| GSE131617 | 183 | 54 | 129 |
| GSE122063 | 100 | 56 | 44 |
| GSE106241 | 60 | 60 | 0 |
| GSE84422_GPL96 | 542 | 328 | 214 |
| GSE84422_GPL97 | 542 | 328 | 214 |
| GSE84422_GPL570 | 62 | 34 | 28 |
| GSE33000 | 467 | 310 | 157 |
| GSE48350 | 253 | 80 | 173 |
| GSE29378 | 63 | 31 | 32 |
| GSE36980 | 80 | 33 | 47 |
| GSE13214 | 76 | 36 | 40 |
| GSE44772 | 690 | 387 | 303 |
| GSE5281 | 161 | 87 | 74 |
| GSE109887 | 78 | 46 | 32 |
|  | 4077 | 2241 | 1836 |

**Supplementary Table2: The primer sequence of target genes (Primer sequence, 5'-3').**

| **Gene** | **Forward** | \| **Reverse** \| \| --- \| |
| --- | --- | --- | --- |
| CCS | GCCTGATTATTGATGAGGGAGAAG | ACAGCAACAGAGCCAAGGTGA |
| CDS2 | GATGATAATCGTGATGTGCGTTC | CCAGGGTGAAGAAGTAATCCGT |
| MYRIP | TCTCGAAGCACCAGCAGTTT | GAGATTGGGCCCTCAGAAGC |
| NRP1 | CCCTCACATTGGGCGTTACTG | ATTCCATGCCCAGAGCTTCC |
| PLEKHA5 | AGAACCTGTGAAAAGAGTGGAC | AGGGTCTATCTTGACCATCCT |
| POLR1D | AAAGAGGGCGATAAGGAACCAG | TTTCGTACTTGTCCTGGCTGC |
| PPP4C | CTGCTGGCACTTAAGGTTCG | ATGATGGCTGACAGGCTGAG |
| GAPDH | GGAAGCTTGTCATCAATGGAAATC | TGATGACCCTTTTGGCTCCC |

**Supplementary Table3: The F-statistic of SNPs for shift work.**

| SNP | F-statistics |
| --- | --- |
| rs666923 | 9.79 |
| rs13019832 | 11.91 |
| rs13009008 | 11.40 |
| rs10932655 | 12.06 |
| rs10865397 | 11.90 |
| rs11922926 | 1.47 |
| rs75814777 | 4.03 |
| rs59815219 | 12.12 |
| rs2087035 | 9.06 |
| rs1375563 | 7.67 |
| rs41534644 | 8.56 |
| rs152603 | 11.69 |
| rs1487441 | 12.07 |
| rs76713680 | 4.37 |
| rs10282168 | 11.56 |
| rs1860826 | 11.57 |
| rs4352868 | 12.00 |
| rs2474711 | 11.30 |
| rs77215157 | 4.76 |
| rs2151875 | 7.56 |
| rs61844343 | 10.70 |
| rs622614 | 9.64 |
| rs34057425 | 11.04 |
| rs1729200 | 9.76 |
| rs1727326 | 8.29 |
| rs11063070 | 7.99 |
| rs12811792 | 8.87 |
| rs4415916 | 3.36 |
| rs9575634 | 8.43 |
| rs150774726 | 2.17 |
| rs55665482 | 5.15 |
| rs28613960 | 12.04 |
| rs950608 | 10.46 |
| rs57885255 | 3.07 |
| rs4808958 | 8.84 |
| rs6039504 | 8.99 |

**Supplementary Table 4: The sensitive analysis of shift work on AD.**

| id.exposure | id.outcome | method | nsnp | b | se | pval |
| --- | --- | --- | --- | --- | --- | --- |
| shift work | Alzheimer's disease | MR Egger | 13 | 3.14 | 2.97 | 0.31 |
| shift work | Alzheimer's disease | Weighted median | 13 | 0.72 | 0.50 | 0.15 |
| shift work | Alzheimer's disease | Simple mode | 13 | 0.86 | 0.80 | 0.30 |
| shift work | Alzheimer's disease | Weighted mode | 13 | 0.96 | 0.85 | 0.28 |

**Supplementary Table 5: The reverse MR analysis (AD on shift work).**

| id.exposure | id.outcome | method | nsnp | b | se | pval | OR |
| --- | --- | --- | --- | --- | --- | --- | --- |
| Alzheimer's disease | shift work | Inverse variance weighted | 40 | -0.003 | 0.003 | 0.337 | 0.997 |

**Supplementary Table 6: The β value of MR analysis result.**

| Gene | β value |
| --- | --- |
| CCS | 0.05 |
| CDS2 | -0.06 |
| MYRIP | -0.55 |
| NRP1 | -0.12 |
| PLEKHA5 | -0.11 |
| POLR1D | 0.08 |
| PPP4C | 0.2 |

**Supplementary Table 7: The result of PCR.**

| ID | CCS | CSD2 | MYRIP | NRP1 | PLEKHA5 | POLRID | PPP4C |
| --- | --- | --- | --- | --- | --- | --- | --- |
| NC1 | 0.989657 | 1.044274 | 1.029897 | 0.933033 | 0.965936 | 0.955945 | 1.026334 |
| NC2 | 0.962594 | 1.029897 | 1.015718 | 1.057018 | 1.086735 | 0.815072 | 0.869043 |
| NC3 | 1.010451 | 0.909093 | 0.896577 | 1.079228 | 0.80107 | 1.090508 | 1.019244 |
| NC4 | 1.038859 | 1.022783 | 1.066216 | 0.939523 | 1.189207 | 1.176907 | 1.099997 |
| AD1 | 1.351911 | 0.928195 | 1.008702 | 0.812252 | 0.598739 | 1.911891 | 1.875792 |
| AD2 | 1.278986 | 0.860055 | 0.947698 | 0.747425 | 0.646176 | 2.020903 | 1.762349 |
| AD3 | 1.333299 | 0.994815 | 0.878126 | 0.678302 | 0.590496 | 1.979313 | 1.726081 |
| AD4 | 1.30586 | 1.015718 | 1.051537 | 0.806642 | 0.532185 | 1.85961 | 1.824499 |

**Supplementary Table 8: The result of ELISA.**

| group | NRP1 | Aβ42 | PPP4C | CCS | ptau181 |
| --- | --- | --- | --- | --- | --- |
| NC | 190.1874 | 26.07239 | 41.71275 | 76.12398 | 13.77976 |
| NC | 167.0997 | 27.70231 | 41.34022 | 87.37687 | 10.38913 |
| NC | 174.8739 | 30.09928 | 37.03208 | 73.3303 | 11.40555 |
| NC | 173.1808 | 25.82983 | 38.66335 | 77.26262 | 12.86105 |
| NC | 149.9886 | 29.85589 | 54.75072 | 68.56605 | 11.89998 |
| NC | 154.6683 | 33.33773 | 50.44347 | 81.06746 | 14.51962 |
| NC | 166.5939 | 29.50827 | 49.99143 | 78.40257 | 10.09081 |
| NC | 177.247 | 30.06451 | 52.32974 | 71.48792 | 12.51533 |
| AD | 156.3428 | 6.622254 | 100.5074 | 96.57567 | 34.87694 |
| AD | 147.6537 | 8.975764 | 67.11998 | 107.175 | 29.9874 |
| AD | 115.4757 | 9.11235 | 64.81514 | 100.2102 | 30.46229 |
| AD | 142.8277 | 13.76604 | 67.27387 | 102.2024 | 33.91613 |
| AD | 131.402 | 29.05652 | 68.92997 | 106.5887 | 21.0076 |
| AD | 117.601 | 21.16174 | 84.21999 | 98.51249 | 13.96079 |
| AD | 128.9288 | 10.68426 | 68.92997 | 95.65693 | 22.16571 |
| AD | 116.7832 | 8.122478 | 88.54939 | 110.2089 | 21.91831 |
| AD | 88.10684 | 12.42958 | 77.45505 | 101.4245 | 44.49256 |
| AD | 97.44542 | 10.5133 | 78.89736 | 96.14038 | 45.26651 |
| AD | 118.7465 | 13.11475 | 67.96671 | 104.9295 | 46.70323 |
| AD | 98.09155 | 22.61203 | 75.23792 | 102.7862 | 48.79344 |
| AD | 86.50248 | 10.47911 | 94.6471 | 100.3073 | 46.96804 |
| AD | 117.7646 | 12.84063 | 87.05132 | 108.7887 | 37.96855 |
| AD | 137.5188 | 23.407 | 94.92529 | 97.59205 | 37.92004 |
| AD | 139.8393 | 8.634373 | 98.27034 | 94.98049 | 11.55529 |

**Supplementary Table 9: The baseline of characteristics of inhouse cohort.**

| Characteristic |  |
| --- | --- |
| onset time | 1.96 ± 1.30 |
| Age | 67.33 ± 9.77 |
| Sex (Female) | 20 (0.44) |
| Left volume | 2.74 ± 0.43 |
| Right volume | 2.82 ± 0.46 |

**Supplementary Table 10: The association between circadian rhythm characteristics and AD biomarkers.**

| **Score** | **AD biomarkers** | **corrlation** | **p value** |
| --- | --- | --- | --- |
| PSQI | left_volume | -0.633446 | 3.01E-06 |
| PSQI | right_volume | -0.6238633 | 4.68E-06 |
| MEQ-SA | left_volume | 0.56260997 | 5.76E-05 |
| MEQ-SA | right_volume | 0.62576542 | 4.29E-06 |
| PSQI | ptau181 | 0.50409385 | 0.0464783 |
| PSQI | Aβ1-42 | -0.6004647 | 0.0139137 |

**STROBE-MR checklist of recommended items to address in reports of Mendelian randomization studies**^1^ ^2^

| **Item No.** | **Section** | **Checklist item** | **Page No.** | **Relevant text from manuscript** |
| --- | --- | --- | --- | --- |
| 1 | **TITLE and ABSTRACT** | Indicate Mendelian randomization (MR) as the study’s design in the title and/or the abstract if that is a main purpose of the study | Page3 | Mendelian randomization (MR) analysis was performed to discover the putative causal effect of shift work for AD. |
|  | **INTRODUCTION** |  |  |  |
| 2 | **Background** | Explain the scientific background and rationale for the reported study. What is the exposure? Is a potential causal relationship between exposure and outcome plausible? Justify why MR is a helpful method to address the study question | Page3 | Previous works have revealed the detrimental effect of shift work on the occurrence and progression of AD. The exposure was the shift work. However, shift work was often accompanied by multiple socioeconomic factors, including poor working conditions, long working hours, low income, increased subjective strains and so on5. Conventional observation studies could not eliminate residual confounding factors and reverse causality completely, which will introduce bias to result. |
| 3 | **Objectives** | State specific objectives clearly, including pre-specified causal hypotheses (if any). State that MR is a method that, under specific assumptions, intends to estimate causal effects | Page3 | Shift work was causally associated with Alzheimer’s disease. Mendelian randomization (MR) analysis was an approach utilizing genetic variants as instrumental variables (IVs) to infer casual association between exposure and outcome6. The inherent properties of genetic variations allocation in MR make it less susceptible to confounding factors, enabling results akin to randomized control trials (RCTs) |
|  | **METHODS** |  |  |  |
| 4 | **Study design and data sources** | Present key elements of the study design early in the article. Consider including a table listing sources of data for all phases of the study. For each data source contributing to the analysis, describe the following: |  |  |
|  | a) | Setting: Describe the study design and the underlying population, if possible. Describe the setting, locations, and relevant dates, including periods of recruitment, exposure, follow-up, and data collection, when available. | Page5 | GWAS summary statistics for shift work was available from UK Biobank involving 263,315 individuals of European ancestry. The summary-level data for clinically diagnosed Alzheimer’s disease (AD) was derived from a large GWAS meta-analysis (GWAS ID: ieu-b-2) with 10,528,610 single nucleotide polymorphisms (SNPs). |
|  | b) | Participants: Give the eligibility criteria, and the sources and methods of selection of participants. Report the sample size, and whether any power or sample size calculations were carried out prior to the main analysis | Page5 | GWAS summary statistics for shift work was available from UK Biobank involving 263,315 individuals of European ancestry. The summary-level data for clinically diagnosed Alzheimer’s disease (AD) was derived from a large GWAS meta-analysis (GWAS ID: ieu-b-2) with 10,528,610 single nucleotide polymorphisms (SNPs). |
|  | c) | Describe measurement, quality control and selection of genetic variants | Supplementary Methods | (i) the selected IVs are strongly associated with exposure factors. (ii) the selected IVs are independent of confounders. (iii) the selected IVs can only act on outcomes through exposure factors. GWAS-significant SNPs (threshold was set to 5×10−6) were pruned by clumping under a stringent window (r2 < 0.001; clumped distance = 10,000 kb) to avoid the linkage disequilibrium (LD). SNPs with minor allele frequency (MAF) less than 0.01 were removed. Furthermore, F-statistic was calculated to assess strength of IVs, and only SNPs with F-statistic more than 10 were retained. |
|  | d) | For each exposure, outcome, and other relevant variables, describe methods of assessment and diagnostic criteria for diseases | Page5 | Shift work was a behaviour that could cause circadian rhythm disruption (CRD), and was defined as “work schedule that falls outside of the normal daytime working hours of 9 AM–5 PM” in UK Biobank. In the original GWAS data of shift work, the frequency was categorized into “Never/Rarely”, “Sometimes”, “Usually” and “Always”, in which “Never/Rarely” was taken as the reference. The summary-level data for clinically diagnosed Alzheimer’s disease (AD) was derived from a large GWAS meta-analysis (GWAS ID: ieu-b-2) with 10,528,610 single nucleotide polymorphisms (SNPs). |
|  | e) | Provide details of ethics committee approval and participant informed consent, if relevant | n/a |  |
| 5 | **Assumptions** | Explicitly state the three core IV assumptions for the main analysis (relevance, independence and exclusion restriction) as well assumptions for any additional or sensitivity analysis | Page7; Supplementary Methods | (i) the selected IVs are strongly associated with exposure factors. (ii) the selected IVs are independent of confounders. (iii) the selected IVs can only act on outcomes through exposure factors. To verify the stability of the results, sensitivity analyses were conducted. Firstly, MR Egger, weighted median, simple mode, and weighted mode were performed if available. Secondly, leave-one-out was utilized to identify the effect on the outcome driven by a single genetic variant. Furthermore, Cochran’s Q statistic was calculated to check heterogeneity between instrumental variables. Egger regression was performed to check directional pleiotropy on outcome and Mendelian Randomization Pleiotropy RESidual Sum and Outlier (MR-PRESSO) test was performed to check overall horizontal pleiotropy among all SNPs. Finally, reverse MR analysis was further performed to avoid the causality effect of outcome on exposure. |
| 6 | **Statistical methods: main analysis** | Describe statistical methods and statistics used |  |  |
|  | a) | Describe how quantitative variables were handled in the analyses (i.e., scale, units, model) | n/a |  |
|  | b) | Describe how genetic variants were handled in the analyses and, if applicable, how their weights were selected | Page5 | GWAS-significant SNPs (threshold was set to 5×10−6) were pruned by clumping under a stringent window (r2 < 0.001; clumped distance = 10,000 kb) to avoid the linkage disequilibrium (LD). SNPs with minor allele frequency (MAF) less than 0.01 were removed. Furthermore, F-statistic was calculated to assess strength of IVs, and only SNPs with F-statistic more than 10 were retained. |
|  | c) | Describe the MR estimator (e.g. two-stage least squares, Wald ratio) and related statistics. Detail the included covariates and, in case of two-sample MR, whether the same covariate set was used for adjustment in the two samples | Page7 | Then the exposure data and outcome data were harmonized and inverse variance weighted (IVW) was utilized to conduct the MR analysis between shift work and AD. MR estimates for single SNP were calculated via Wald ratio and IVW was performed in the case of more than one SNP available. |
|  | d) | Explain how missing data were addressed | n/a |  |
|  | e) | If applicable, indicate how multiple testing was addressed | Page10 | False discovery rate (FDR) adjustment was utilized for multiple comparison corrections. |
| 7 | **Assessment of assumptions** | Describe any methods or prior knowledge used to assess the assumptions or justify their validity | Page7 | To verify the stability of the results, sensitivity analyses were conducted. Firstly, MR Egger, weighted median, simple mode, and weighted mode were performed if available. Secondly, leave-one-out was utilized to identify the effect on the outcome driven by a single genetic variant. Furthermore, Cochran’s Q statistic was calculated to check heterogeneity between instrumental variables. Egger regression was performed to check directional pleiotropy on outcome and Mendelian Randomization Pleiotropy RESidual Sum and Outlier (MR-PRESSO) test was performed to check overall horizontal pleiotropy among all SNPs. Finally, reverse MR analysis was further performed to avoid the causality effect of outcome on exposure. |
| 8 | **Sensitivity analyses and additional analyses** | Describe any sensitivity analyses or additional analyses performed (e.g. comparison of effect estimates from different approaches, independent replication, bias analytic techniques, validation of instruments, simulations) | Page7 | To verify the stability of the results, sensitivity analyses were conducted. Firstly, MR Egger, weighted median, simple mode, and weighted mode were performed if available. Secondly, leave-one-out was utilized to identify the effect on the outcome driven by a single genetic variant. Furthermore, Cochran’s Q statistic was calculated to check heterogeneity between instrumental variables. Egger regression was performed to check directional pleiotropy on outcome and Mendelian Randomization Pleiotropy RESidual Sum and Outlier (MR-PRESSO) test was performed to check overall horizontal pleiotropy among all SNPs. Finally, reverse MR analysis was further performed to avoid the causality effect of outcome on exposure. |
| 9 | **Software and pre-registration** |  |  |  |
|  | a) | Name statistical software and package(s), including version and settings used | Page10 | All data processing and analysis were performed in R 4.3.1 software. The mendelian randomization was conducted via the TwoSampleMR package. |
|  | b) | State whether the study protocol and details were pre-registered (as well as when and where) | n/a |  |
|  | **RESULTS** |  |  |  |
| 10 | **Descriptive data** |  |  |  |
|  | a) | Report the numbers of individuals at each stage of included studies and reasons for exclusion. Consider use of a flow diagram | n/a |  |
|  | b) | Report summary statistics for phenotypic exposure(s), outcome(s), and other relevant variables (e.g. means, SDs, proportions) | Supplementary Table |  |
|  | c) | If the data sources include meta-analyses of previous studies, provide the assessments of heterogeneity across these studies | n/a |  |
|  | d) | For two-sample MR:  i.  Provide justification of the similarity of the genetic variant-exposure associations between the exposure and outcome samples  ii.  Provide information on the number of individuals who overlap between the exposure and outcome studies | Supplementary Table |  |
| 11 | **Main results** |  |  |  |
|  | a) | Report the associations between genetic variant and exposure, and between genetic variant and outcome, preferably on an interpretable scale | Supplementary Table |  |
|  | b) | Report MR estimates of the relationship between exposure and outcome, and the measures of uncertainty from the MR analysis, on an interpretable scale, such as odds ratio or relative risk per SD difference | Page10-11 | Results of mendelian randomization showed that shift work was causally associated with AD [odds ratio (OR) = 2.49, 95% CI = 1.79 - 3.19, p = 0.01, method = IVW] |
|  | c) | If relevant, consider translating estimates of relative risk into absolute risk for a meaningful time period | n/a |  |
|  | d) | Consider plots to visualize results (e.g. forest plot, scatterplot of associations between genetic variants and outcome versus between genetic variants and exposure) | Figure2 | The effect of each single SNP was demonstrated in Figure 2D. To investigate and correct pleiotropy for the outcome, we conducted Egger regression and MR-PRESSO. As demonstrated in Figure 2E, no directional and horizontal pleiotropy was found (p = 0.47, method = MR Egger; no outlier was found, method = weighted median). The heterogeneity was tested via Cochran’s Q statistic and no heterogeneity was observed (p = 0.34, method = MR Egger; p = 0.37, method = IVW) (Figure 2E). |
| 12 | **Assessment of assumptions** |  |  |  |
|  | a) | Report the assessment of the validity of the assumptions | Page10 | The sensitive analysis consistently indicated a suggestive and positive causal association with AD, with detailed information provided in Supplementary Table 4. |
|  | b) | Report any additional statistics (e.g., assessments of heterogeneity across genetic variants, such as *I^2^*, Q statistic or E-value) | Page10-11 | To investigate and correct pleiotropy for the outcome, we conducted Egger regression and MR-PRESSO. As demonstrated in Figure 2E, no directional and horizontal pleiotropy was found (p = 0.47, method = MR Egger; no outlier was found, method = weighted median). The heterogeneity was tested via Cochran’s Q statistic and no heterogeneity was observed (p = 0.34, method = MR Egger; p = 0.37, method = IVW) (Figure 2E). |
| 13 | **Sensitivity analyses and additional analyses** |  |  |  |
|  | a) | Report any sensitivity analyses to assess the robustness of the main results to violations of the assumptions | Page10; Supplementary Table | The sensitive analysis consistently indicated a suggestive and positive causal association with AD, with detailed information provided in Supplementary Table 4. |
|  | b) | Report results from other sensitivity analyses or additional analyses | Supplementary Table | The sensitive analysis consistently indicated a suggestive and positive causal association with AD, with detailed information provided in Supplementary Table 4. |
|  | c) | Report any assessment of direction of causal relationship (e.g., bidirectional MR) | Page11 | Finally, the MR analysis of AD on shift work was performed to avoid a reverse causal effect, and no evidence was identified for the causal effect of AD on shift work (OR = 0.997, 95% CI = 0.991 - 1.003, p = 0.34, method = IVW) (Supplementary Table 5). |
|  | d) | When relevant, report and compare with estimates from non-MR analyses | n/a |  |
|  | e) | Consider additional plots to visualize results (e.g., leave-one-out analyses) | Page10 | The effect of each single SNP was demonstrated in Figure 2D. |
|  | **DISCUSSION** |  |  |  |
| 14 | **Key results** | Summarize key results with reference to study objectives | Page14-15 | By applying mendelian randomization (MR) analysis, we found a causal, detrimental effect of shift work on Alzheimer’s disease (AD). |
| 15 | **Limitations** | Discuss limitations of the study, taking into account the validity of the IV assumptions, other sources of potential bias, and imprecision. Discuss both direction and magnitude of any potential bias and any efforts to address them | Page19-20 | Firstly, the MR analysis between shift work and AD was confined to Europeans, as the GWAS data for other ethnicities were not well developed. The occurrence of AD was influenced, in part, by genetic factors, and these influences may vary to some extent across different ethnic groups. Nevertheless, the impact of shift work on populations appeared to be widespread. Therefore, it was imperative to replicate these analyses in other ethnicities to accurately capture the influence of shift work on the development of AD across diverse ethnic backgrounds. |
| 16 | **Interpretation** |  |  |  |
|  | a) | Meaning: Give a cautious overall interpretation of results in the context of their limitations and in comparison with other studies | Page20 | In the present study, we revealed the causal association between shift work and Alzheimer's disease (AD) through Mendelian randomization (MR) analysis, highlighting that shift work has a detrimental impact on the occurrence of AD. |
|  | b) | Mechanism: Discuss underlying biological mechanisms that could drive a potential causal relationship between the investigated exposure and the outcome, and whether the gene-environment equivalence assumption is reasonable. Use causal language carefully, clarifying that IV estimates may provide causal effects only under certain assumptions | Page15;  Supplementary material | We also identified seven putative causal circadian-related genes for AD, shedding light on the underlying mechanism connecting gene expression influence by shift work to the development of AD. |
|  | c) | Clinical relevance: Discuss whether the results have clinical or public policy relevance, and to what extent they inform effect sizes of possible interventions | Page20 | The current research was the first to combine the transcriptome with Genome-Wide Association Study (GWAS) data to explore circadian rhythm-disrupting behavior and AD, which demonstrated public health significance in the realm of AD prevention and offered a broad biological and clinical perspective for future therapeutic interventions. |
| 17 | **Generalizability** | Discuss the generalizability of the study results (a) to other populations, (b) across other exposure periods/timings, and (c) across other levels of exposure | Page19-20 | Firstly, the MR analysis between shift work and AD was confined to Europeans, as the GWAS data for other ethnicities were not well developed. However, tThe occurrence of AD was influenced, in part, by genetic factors, and these influences may vary to some extent across different ethnic groups. Nevertheless, the impact of shift work on populations appeared to be widespread. Therefore, it was imperative to replicate these analyses in other ethnicities to accurately capture the influence of shift work on the development of AD across diverse ethnic backgrounds. |
|  | **OTHER INFORMATION** |  |  |  |
| 18 | **Funding** | Describe sources of funding and the role of funders in the present study and, if applicable, sources of funding for the databases and original study or studies on which the present study is based | Page21-22 | This work was supported by Henan Province Medical Science and Technology Research Program Joint Construction Project (LHGJ20220332). |
| 19 | **Data and data sharing** | Provide the data used to perform all analyses or report where and how the data can be accessed, and reference these sources in the article. Provide the statistical code needed to reproduce the results in the article, or report whether the code is publicly accessible and if so, where | Page21 | Public datasets included in the present work were from GEO, IEU Open GWAS project, UK Biobank, and eQTLGen. |
| 20 | **Conflicts of Interest** | All authors should declare all potential conflicts of interest | Page21 | The authors declare that they have no competing interests. |

This checklist is copyrighted by the Equator Network under the Creative Commons Attribution 3.0 Unported (CC BY 3.0) license.

1. Skrivankova VW, Richmond RC, Woolf BAR, Yarmolinsky J, Davies NM, Swanson SA, et al. Strengthening the Reporting of Observational Studies in Epidemiology using Mendelian Randomization (STROBE-MR) Statement. JAMA. 2021;under review.

2. Skrivankova VW, Richmond RC, Woolf BAR, Davies NM, Swanson SA, VanderWeele TJ, et al. Strengthening the Reporting of Observational Studies in Epidemiology using Mendelian Randomisation (STROBE-MR): Explanation and Elaboration. BMJ. 2021;375:n2233.
